# Determination of electrostatic force and its characteristics based on phase difference by amplitude modulation atomic force microscopy

**DOI:** 10.1186/s11671-016-1765-2

**Published:** 2016-12-12

**Authors:** Kesheng Wang, Jia Cheng, Shiji Yao, Yijia Lu, Linhong Ji, Dengfeng Xu

**Affiliations:** State Key Laboratory of Tribology, Tsinghua University, Beijing, 100084 China

**Keywords:** Electrostatic force, Amplitude modulation atomic force microscopy (AM-AFM), Phase difference, Tip-sample distance, Applied voltage

## Abstract

Electrostatic force measurement at the micro/nano scale is of great significance in science and engineering. In this paper, a reasonable way of applying voltage is put forward by taking an electrostatic chuck in a real integrated circuit manufacturing process as a sample, applying voltage in the probe and the sample electrode, respectively, and comparing the measurement effect of the probe oscillation phase difference by amplitude modulation atomic force microscopy. Based on the phase difference obtained from the experiment, the quantitative dependence of the absolute magnitude of the electrostatic force on the tip-sample distance and applied voltage is established by means of theoretical analysis and numerical simulation. The results show that the varying characteristics of the electrostatic force with the distance and voltage at the micro/nano scale are similar to those at the macroscopic scale. Electrostatic force gradually decays with increasing distance. Electrostatic force is basically proportional to the square of applied voltage. Meanwhile, the applicable conditions of the above laws are discussed. In addition, a comparison of the results in this paper with the results of the energy dissipation method shows the two are consistent in general. The error decreases with increasing distance, and the effect of voltage on the error is small.

## Background

Electrostatic force is one of the basic forces in nature. Accurate measurement of electrostatic force at the micro/nano scale contributes to research of the charge distribution near donors in semiconductors, radiation effects in organic photovoltaic cells, bond position in molecules, and other scientific problems, as well as exploring smaller forces such as the Casimir force and gravitation; it can also be used to verify the applicability of certain macroscopic laws to the micro/nano scale. Also, the measurement of electrostatic force plays an important role in micro/nano devices or large-scale integrated circuit equipment [1–4]. Because of the micro/nano scale effect, electrostatic force becomes non-negligible and directly affects the relative motion between devices. Electrostatic force must be determined to realize precise regulation and control of device performance. Therefore, the measurement of microcosmic electrostatic force is of great scientific value and engineering significance. However, in general, electrostatic force cannot be directly measured and can only be obtained by some indirect methods.

Atomic force microscopy (AFM), as one of the most powerful scientific research tools, has broad application prospects in the detection of micro force. However, there are still no mature standard methods to measure electrostatic force by AFM. A novel method with high accuracy has been reported recently [5]. Electrostatic force can be calculated based on the principle of energy dissipation, without any approximate treatment used, but there are very stringent requirements for experimental conditions, and ultrahigh vacuum and ultrahigh pulse environments are required to ensure a very high signal-to-noise ratio. Any small disturbance will have a great impact on the measurement results. There will be difficulties in realization and complexities in operation.

In terms of feedback parameters, AFM can be divided into two modes: amplitude modulation atomic force microscopy (AM-AFM) and frequency modulation atomic force microscopy (FM-AFM), where AM-AFM has an additional feature; it enables the acquisition of the phase difference image of probe oscillation as well as the sample surface topography [6, 7]. The variation of electrostatic force between the probe and the sample will cause a change in the probe resonance characteristics, shown as a phase difference, directly reflecting the electrostatic force contrast, i.e., the relative magnitude of the electrostatic force. However, the absolute magnitude of electrostatic force cannot be obtained.

Phase difference measurement experiments are not limited by the external environment and are easy to perform in both vacuum and atmosphere [8]. Consequently, a possible research direction is obtaining the specific electrostatic force according to the phase difference information. Some scholars have carried out relevant research, obtaining electrostatic force gradient images through the phase difference [9, 10], but the absolute magnitude of the electrostatic force cannot be acquired. A mathematical model of the electrostatic force gradient has been established [9], but it conforms well to the experimental results only for a very small tip-sample distance. The degree of coincidence gets worse with increasing distance, and the range of application is narrow. The variation laws of the phase difference of vanadium-pentoxide nanowire samples with tip-sample distance and applied voltage have been investigated [11], but the results are not connected with the electrostatic force gradient. Finally, the absolute magnitude of the electrostatic force still was not obtained. Electrostatic force can be calculated based on phase difference measurement, combined with finite element simulation [12], but the sample is a metal wire with discontinuous material, and the voltage is only selectively applied on a local part of the sample, so there are some limitations in the material and structure of the sample and the way of applying voltage. Moreover, the effect of voltage on the electrostatic force has not been studied.

In this paper, we first determine a reasonable way of applying voltage, based on the measurement results of the phase difference in AM-AFM by taking the electrostatic chuck in an integrated circuit manufacturing process as a sample and applying the same voltage, respectively, on the probe and the sample electrode. Then, the phase difference is converted into an electrostatic force gradient according to certain transformational relationships. The relationship between electrostatic force and tip-sample distance can be obtained by simulating the boundary value of electrostatic force using COMSOL software. We alter the applied voltage to observe the influence of voltage on electrostatic force. Finally, the results in this paper are compared with the results of the energy dissipation method.

## Methods

### Fundamental principles

The difference between the oscillation phase of a probe in the non-resonance region and the free oscillation phase in atmosphere is [13–15]:

1 $$ \varDelta \varphi \approx -{ \tan}^{-1}\left(\ \frac{Q}{k}\cdot \frac{\partial F}{\partial z}\ \right) $$

where *F* is the electrostatic force between probe and sample, *z* is the tip-sample distance, *k* is the spring constant of the cantilever, and *Q* is the quality factor of the cantilever. Note that *F* is actually the sum of electrostatic force and Van der Waals’ force, but Van der Waals’ force can be neglected within the range of the distances involved in this study.

We yield from Eq. (1):

2 $$ \frac{\partial F}{\partial z}\approx -\frac{k}{Q} \tan \left(\varDelta \varphi \right) $$

Equation (2) is just an expression of the electrostatic force gradient, and corresponding treatment is necessary to obtain the electrostatic force.

The tip can be simplified as a combination model of a cone and a spherical cap when the radius of the tip apex is small relative to the distance *z* [16–20]. Based on this model, the variation of the electrostatic force gradient with distance can be assumed to be in the form [12]:

3 $$ \frac{\partial F}{\partial z}=a+\frac{b}{z+c} $$

where *a*, *b*, and *c* are undetermined constants that can be obtained by fitting the experimental data according to the minimum error principle.

Integrating the electrostatic force gradient leads to the electrostatic force at any distance *z*:

4 $$ F={\displaystyle {\int}_z^{+\infty }}\ \frac{\partial F}{\partial z}\mathrm{d}z={\displaystyle {\int}_z^{z_{\max }}}\ \frac{\partial F}{\partial z}\mathrm{d}z + {\displaystyle {\int}_{z_{\max}}^{+\infty }}\ \frac{\partial F}{\partial z}\mathrm{d}z $$

where* z*_max_ is the maximum distance set in the experiment.

It must be pointed out that the tip-sample distance should vary within a certain range. If the distance is too small, the electrostatic force is so strong that the tip tends to be attracted to the sample surface, leading to a large error in the experimental results. If the distance is too large, the signal-to-noise ratio will be poor and noise interference cannot be ignored, making it difficult to accurately identify real phase information.

That is:

5 $$ {F}_{z_{\max }}={\displaystyle {\int}_{z_{\max}}^{+\infty }}\ \frac{\partial F}{\partial z}\mathrm{d}z $$

where ${F}_{z_{\max }}$ is the electrostatic force at the maximum distance.

Substituting Eq. (3) and Eq. (5) into Eq. (4) yields:

6 $$ F={\displaystyle {\int}_z^{z_{\max }}}\left(a+\frac{b}{z+c}\right)\mathrm{d}z + {F}_{z_{\max }}=a\ \left({z}_{\max }-z\right) + b\  \ln \frac{z_{\max }+c}{z+c} + {F}_{z_{\max }} $$

The boundary value ${F}_{z_{\max }}$ in Eq. (6) can be obtained using the following equation [17, 21, 22]:

7 $$ {F}_{z_{\max }}={\displaystyle \underset{S}{\int }}\frac{1}{2}{\varepsilon}_0{E}_z^2\mathrm{d}S $$

where *S* is the probe surface area under the action of the electrostatic force, *ε*_0_ is the vacuum dielectric constant, and *E*_*z*_ is the electric field intensity distribution along the *z* direction. It is usually difficult to solve Eq. (7) by analytical means. In practical application, numerical simulation is generally used for calculation.

### Experimental equipment

**Fig. 1:**
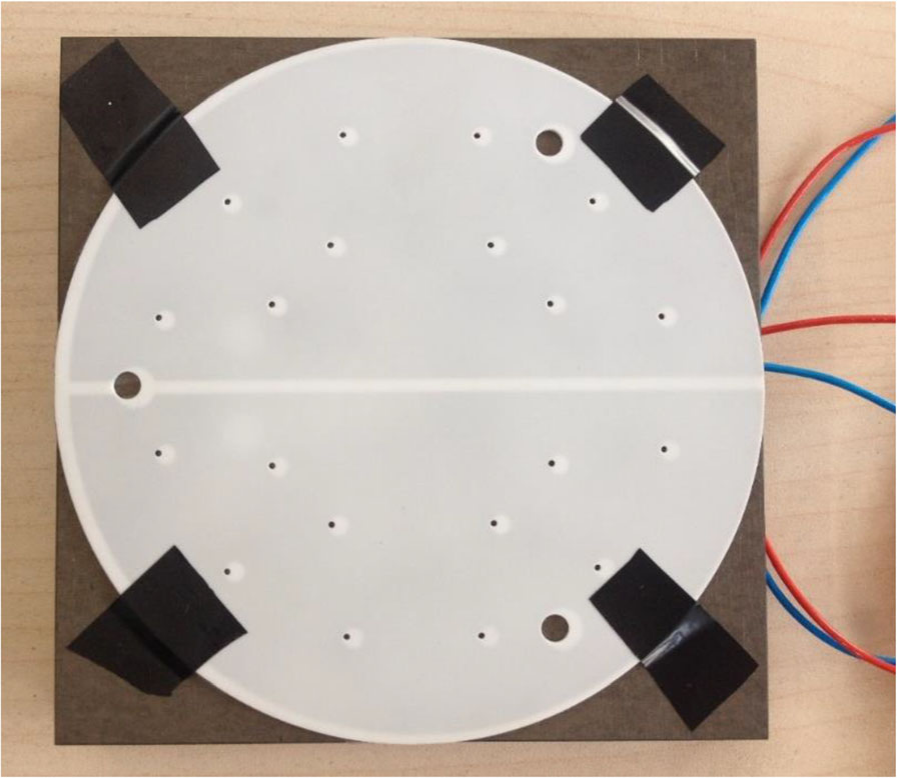
Sample

The sample prepared for the experiment is made of alumina ceramic with a diameter of 100 mm and a thickness of 1.02 mm, as shown in Fig. 1. Two half round (“double D” type) tungsten electrodes are embedded in the sample. The distance between the electrodes and the upper surface of the sample is 0.3 mm. There are many small holes on the sample surface. In a real integrated circuit manufacturing process, the sample is an important part of the electrostatic chuck. Backside gas for heat conduction and temperature control of the wafer will outflow from the above mentioned small holes. In addition, three large holes on the sample surface are pin holes. After a process cycle is completed, pins extend out of the holes, lifting up the wafer for it to be removed. Under the sample, there is a hollow base where the lead wires protrude for connecting to an external power supply. The sample is fixed on the base with adhesive tape so that the sample cannot move relative to the base.

The model number of the selected probe is OMCL-AC240TS-R3, produced by Olympus Company, Japan. The probe is made of silicon. The cantilever is 240-μm long, 40-μm wide, and 2.4-μm thick. The tip is 14-μm high, and the radius at the tip apex is 7 nm. The half angle of the tip body is 17.5°. The spring constant of the cantilever is 2 N/m, and the resonance frequency is 70 kHz. The experimental condition is atmospheric environment, and the quality factor is 145. The selected AFM is the product of the US Bruker’ Dimension Icon, consisting of an electrostatic force module (EFM). The resolution in the *z* direction is 0.06 nm. A common DC power supply with a range of 15 V was used to provide the voltage for the electrode of the sample. In addition, the AFM itself can also apply voltage on the probe via software operation interface. The applied voltage limit required by the AFM is no higher than 10 V.

## Results and discussions

**Fig. 2:**
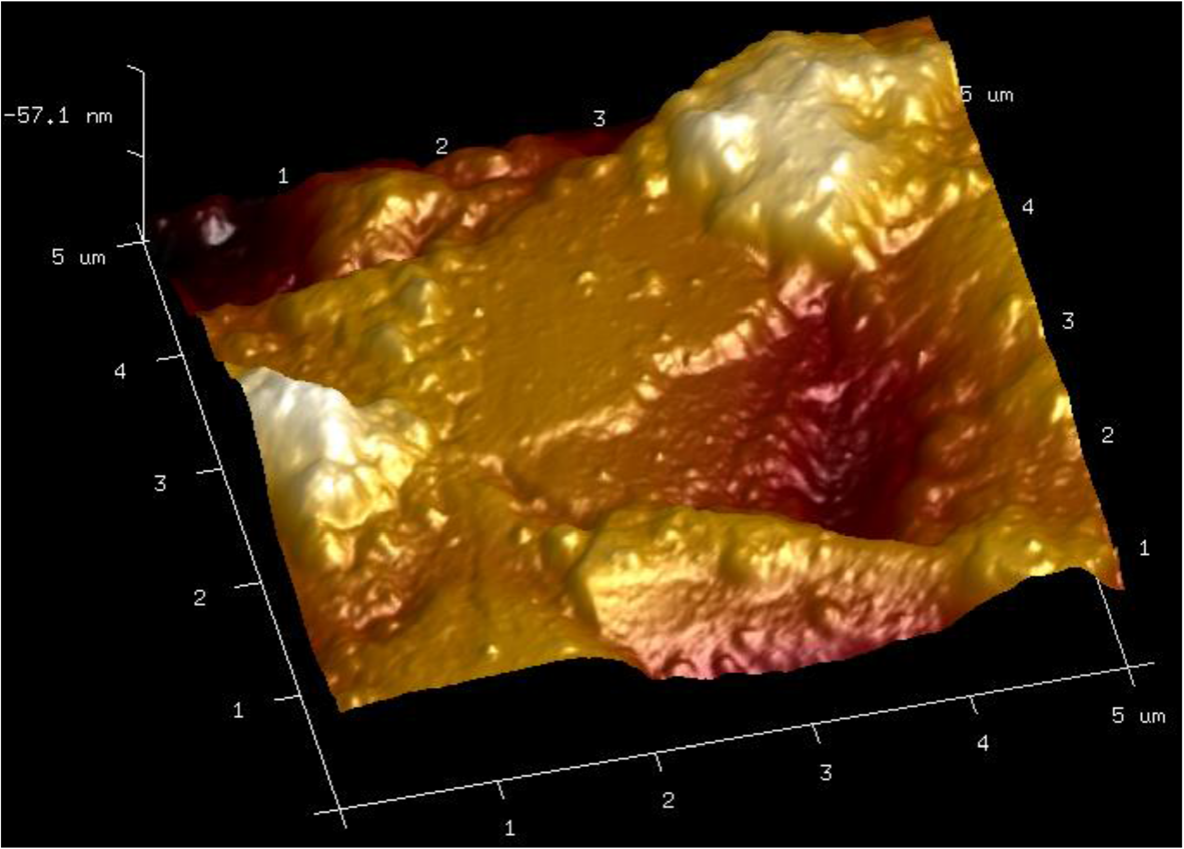
Sample surface topography obtained by AM-AFM

**Fig. 3:**
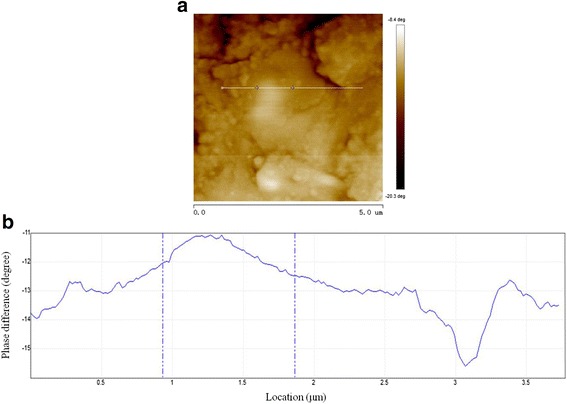
Phase difference information obtained by AM-AFM. **a** Phase difference distribution within square area. **b** Variation of phase difference along the selected horizontal line

The surface topography image and phase difference information of the sample can be obtained simultaneously by means of the EFM module in AM-AFM. A three-dimensional image of the sample surface topography within a scanning area of 5 μm × 5 μm is shown in Fig. 2. The phase difference distribution within the same area is shown in Fig. 3a, and the variation of phase difference along an arbitrary horizontal line is shown in Fig. 3b. Thus, the phase difference corresponding to each point on the sample can be determined.

### No applied voltage on the probe and the sample

**Fig. 4:**
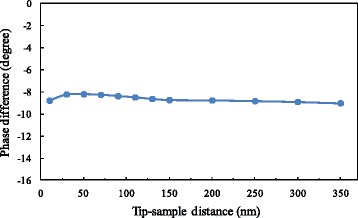
Variation of phase difference with distance without applied voltage

Before the formal experiment, it was necessary to calibrate the phase difference. The phase difference without applied voltage was measured as the initial offset. The variation range of the tip-sample distance was set as 10–350 nm, where a data point was taken at an interval of 20 nm within the range of 10–150 nm, and a data point was taken at an interval of 50 nm within the range of 150–350 nm. In the experiment, at first, the tip was lifted to the highest position and the corresponding phase difference was recorded. Then, the tip was gradually lowered to the lowest position and the corresponding phase difference was recorded in turn. The variation of phase difference with distance is shown in Fig. 4. Although there was no applied voltage on the probe and the sample, the phase difference is not zero. This is because even under a natural state there is inevitably surface potential for the sample, forming an initial potential difference between the sample surface and the probe. The comprehensive effects of this electrostatic field and Van der Waals’ force lead to the existence of a non-zero phase difference. We find that the variation of the measured initial phase difference with distance is not obvious and is basically stable in the range from −8° to −9°.

### Applied voltage on the electrode of the sample

**Fig. 5:**
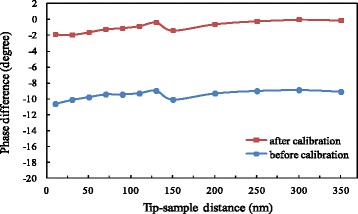
Variation of phase difference with distance at 5 V applied on sample electrode

The electrode embedded in the sample was connected to an external DC power supply and a voltage of 5 V was applied. The phase differences at various distances were in turn measured in the order of tip position from high to low. According to the experimental results in Fig. 4, a calibration was conducted to exclude the contributions of surface potential difference and Van der Waals’ force. The results before calibration and after calibration are shown in Fig. 5. The phase difference after calibration is near zero, and the variation of the phase difference with tip-sample distance is small. The above experiment was repeated by horizontally moving the probe and changing the horizontal relative position between the probe and the sample. Still, a similar conclusion was reached. Moreover, when we increased the applied voltage to 10 V, there was still no significant change in the results. The above phenomena show the small effect of measuring the phase difference by applying voltage on the electrode of the sample.

For these reasons, on the one hand, the applied voltage may be still not high enough. Not enough charges can be polarized on the sample surface to generate an electrostatic force that can be detected by AFM. Nevertheless, the voltage cannot be increased again because the external voltage of the AFM used in the experiment is required to be no more than 10 V. On the other hand, there are fewer induced charges on the probe. Because the voltage is applied on the electrode of the sample, the polarized charges are distributed on almost the whole surface of the sample. Thus, there are few charges concentrated near the probe, resulting in an electrostatic force between the probe and the sample that is too weak to generate a phase difference. At the same time, we used COMSOL software to conduct the numerical simulations. The voltage of 5 and 10 V was respectively applied on the electrode of the sample. The computational results show that induced charge density on the probe is zero for both cases, thus confirming the reasonableness of the above explanations.

### Applied voltage on the probe

**Fig. 6:**
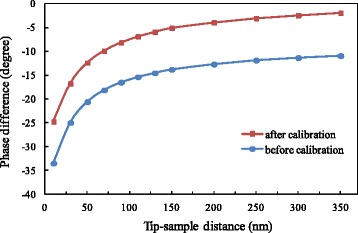
Variation of phase difference with distance at 5 V applied on probe

The AFM built-in system was used to apply voltage on the probe. The voltage was set at 5 V. The phase differences at various distances were in turn measured as the tip was gradually lowered. According to the results in Fig. 4, a calibration was carried out to exclude the contributions of surface potential difference and Van der Waals’ force. The results before calibration and after calibration are shown in Fig. 6. It can be observed that the variation of the phase difference after calibration becomes significant with increasing distance. At the minimum distance, the phase difference is approximately −25°, while the phase difference is close to zero at the maximum distance.

**Fig. 7:**
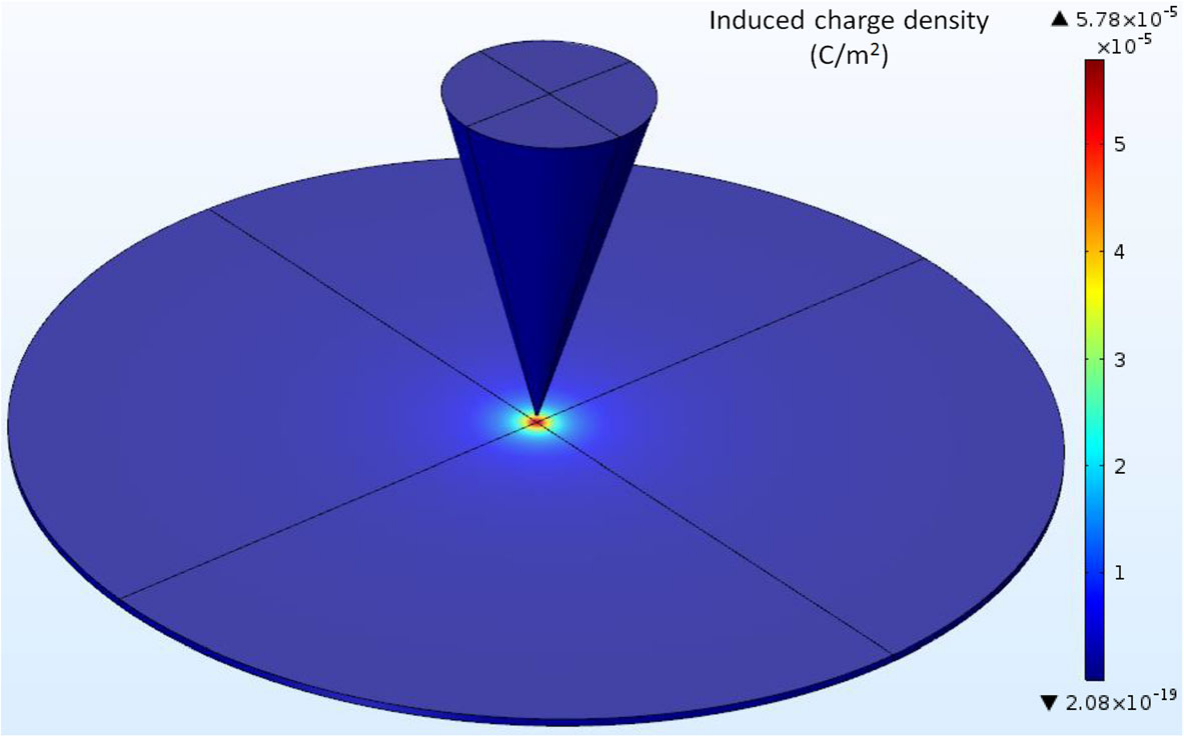
Distribution of induced charge density on sample surface at 350 nm under 5 V

**Fig. 8:**
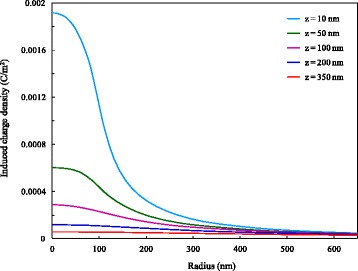
Variation of induced charge density on sample surface in radial direction at different distances under 5 V

It can be seen that applying the same voltage on the probe or on the electrode of the sample leads to very different effects. This occurs because when we apply voltage on the probe, the induced charges can actually be produced only on the local area of the sample surface corresponding to the probe position, while there are relatively few induced charges on other areas of the sample surface. The electrostatic force generated by highly concentrated induced charges is sufficient to cause an obvious phase difference. Similarly, COMSOL software was utilized to carry out the numerical simulations. By applying the voltage of 5 V on the probe, we obtained the distribution of induced charge density on the sample surface at different distances. The result for the distance of 350 nm is shown in Fig. 7. It should be noted that the real diameter of the sample is too large relative to the size of the probe, and after a preliminary simulation, we found that there was almost zero charge induced on the sample surface far from the probe. In order to simplify the model and to facilitate the observation of simulation results, we only selected the range of 40-μm diameter on the sample surface as the computational domain. It can be seen from Fig. 7 that the induced charge density is distributed in the mode of concentric circles, centered at the projection point of the tip apex on the sample surface, and decreases gradually along the radial direction. We plotted the variation curve of induced charge density in radial direction, as shown in Fig. 8. Obviously, the smaller the distance is, the larger the maximum value of induced charge density is, but the faster the induced charge density decays. After the radius exceeds 600 nm, the curves corresponding to different distances tend to be steady and are approximately coincident. When the distances are 10, 50, 100, 200, and 350 nm, the induced charge density at the center is 37.3, 13.3, 7.0, 3.3, and 2.0 times as much as that at the radius of 600 nm, respectively. The multiple and the decreasing rate of induced charge density together reflect the degree of the concentration or non-uniformity of the induced charges in the radial direction, and therefore can be used as the assessment indicators of the local effect of induced charges. The smaller the distance is, the larger the multiple and the decreasing rate of induced charge density are, the stronger the local effect is. As the distance increases, the local effect will be weakened gradually. As a result, the above variation characteristics are found in the phase difference.

Therefore, to obtain effective experimental results, the way of applying voltage to the probe should be adopted. If there is no special note later in this paper, the voltage will be assumed to be applied on the probe.

**Fig. 9:**
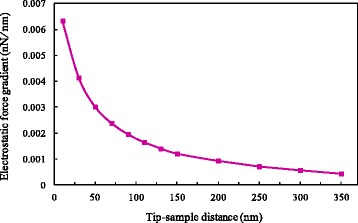
Variation of electrostatic force gradient with distance at 5 V applied on probe

By substituting the phase differences at various distances into Eq. (2), a curve of electrostatic force gradient with distance can be obtained, as shown in Fig. 9. Using Eq. (3), three undetermined constants are respectively obtained by means of the least squares method as follows: *a* = − 0.000204 nN/nm, *b* = 0.256325 nN, and *c* = 29.161974 nm.

**Fig. 10:**
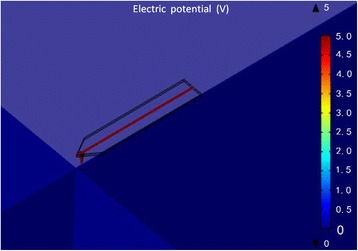
Simulation for surface potential of probe and sample at 350 nm under 5 V

Next, numerical simulation was employed to calculate the absolute magnitude of electrostatic force at the maximum distance. A three-dimensional model of the probe and the sample was established. As described previously, the tip can be simplified as a combination of a cone and a spherical cap, and the distance between the tip apex and the sample was set at 350 nm. As the probe is very small relative to the sample, the densest mesh was selected. The steady-state electric potential distributions of the probe and the sample surface with an applied voltage of 5 V are shown in Fig. 10. The electric field intensity along the *z* direction is automatically converted by the AC/DC module in COMSOL software. Based on Eq. (7), the integral expression is set in the software to obtain the boundary value of the electrostatic force as follows: ${F}_{z_{\max }}=0.0672\ \mathrm{n}\mathrm{N}$.

Substituting the values of *a*, *b*, *c*, and ${F}_{z_{\max }}$ into Eq. (6) yields:

8 $$ F= - 0.000204\ \left(350-z\right)+0.256325\  \ln \frac{350+29.161974}{z+29.161974}+0.0672 = 0.000204\ z-0.256325\  \ln \left(z+29.161974\right)+1.517848 $$

Equation (8) represents the absolute magnitude of the electrostatic force as a function of tip-sample distance under an applied voltage of 5 V. Note that the units of *F* and *z* are nN and nm, respectively.

**Fig. 11:**
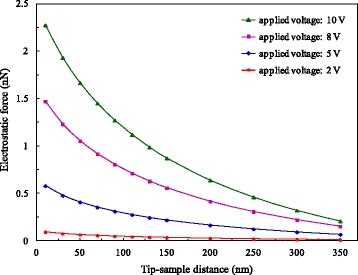
Variation of electrostatic force with distance under different voltages

We also performed the measurements under the applied voltages of 2, 8, and 10 V. The experimental condition, procedure, and data processing method are identical to those under 5 V. Therefore, the absolute magnitude of the electrostatic force as a function of tip-sample distance at different voltages can be derived, and the corresponding curves are shown in Fig. 11. The electrostatic force decreases with increasing distance, and the higher the voltage is, the faster the electrostatic force decreases. As the distance increases gradually, the electrostatic force tends to be stable. The variation of electrostatic force can be neglected for distances greater than 350 nm.

**Fig. 12:**
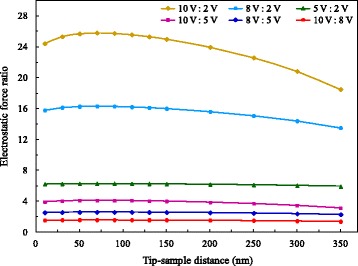
Ratios of electrostatic forces under different voltages

The quantitative comparisons of the electrostatic forces under two different voltages are shown in Fig. 12. We note that the ratio of any two electrostatic forces is approximately equal to the square of the ratio of their corresponding voltages. The smaller the voltage ratio is, the clearer this law is. As the voltage ratio increases, the deviation is magnified gradually and the curve slope becomes larger. On the whole, when the tip-sample distance varies from 10 to 200 nm, the above quantitative relationship can be well satisfied. When the distance exceeds 200 nm, the electrostatic force ratio decreases gradually and the deviation shows an enlarging trend. We take the case of the uppermost curve in Fig. 12, which has the largest curvature among the six curves. The calculation indicates that the electrostatic force ratio at a distance of 200 nm is only 4.2% less than the standard value of 25, while the one at a distance of 350 nm is 26.0% less than the same standard value.

The above results reflect the variation characteristics of electrostatic force under applied voltage, i.e., within a certain range of tip-sample distance, electrostatic force is proportional to the square of the applied voltage. Later, nonlinear effects began to appear with increasing distance, and the influence of voltage on the electrostatic force is gradually weakened. Obviously, the relationship between electrostatic force and voltage observed at the micro/nano scale is remarkably similar to the characteristics at the macroscopic scale [23].

### Comparison with the results of energy dissipation method

We take the results of the energy dissipation method mentioned in the introduction as a reference standard to verify the accuracy of the results reported in this paper. It should be pointed out that only experimental data within a distance of 25 nm were provided by the literature [5], so we can only compare them within this range. The comparisons under 5 and 10 V are shown in Table 1 and Table 2, respectively.

**Table 1:** Comparison of electrostatic forces obtained by the two methods under 5 V

Tip-sample distance (nm)	Electrostatic force obtained by energy dissipation method [5] (nN)	Electrostatic force obtained in this paper (nN)	Error
10	0.833	0.57976	−30.40%
15	0.667	0.54998	−17.54%
20	0.583	0.52351	−10.20%
25	0.522	0.49970	−4.27%

**Table 2:** Comparison of electrostatic forces obtained by the two methods under 10 V

Tip-sample distance (nm)	Electrostatic force obtained by energy dissipation method [5] (nN)	Electrostatic force obtained in this paper (nN)	Error
10	3.332	2.27661	−31.67%
15	2.668	2.18106	−18.25%
20	2.332	2.09247	−10.27%
25	2.088	2.00994	−3.74%

From Table 1 and Table 2, it can be seen that the electrostatic force obtained in this paper is generally smaller than that obtained by the energy dissipation method within the comparable range, but the error is remarkably reduced with the increasing tip-sample distance. Furthermore, the errors corresponding to the same distance show little dependence on the applied voltage. The average errors under the two voltages are also similar, 15.6 and 16.0%, respectively.

The error source can be analyzed as follows. On the one hand, the interaction forces between the tip and the sample include, in addition to the electrostatic force, other forms of force, such as Van der Waals’ force. The influence of these forces is not negligible for quite small distances. However, compared with the long-range electrostatic force, their effective ranges are short. These forces decay rapidly with increasing distance and decrease faster than the electrostatic force, making the electrostatic force gradually become predominant [21, 24–29]. Therefore, the larger the distance is, the smaller the error is. According to this law, it can be predicted that when the distance is beyond 25 nm, the error will tend to be stable, without large fluctuations. This means that the results obtained by the two methods are generally consistent within the variation range of the distance involved in this study. On the other hand, for the method adopted in this study, there is some approximation of the theoretical formula itself, and a simplified model is used to simulate the boundary value of electrostatic force, which will inevitably affect the accuracy of the results.

The main advantages of the method in this paper are fewer restrictions on experimental conditions, relatively low experimental cost, strong practicability, and good repeatability. Under the premise of modest requirements for the measurement accuracy of the electrostatic force at the micro/nano scale, this method can replace the energy dissipation method and has a great application potential.

## Conclusions

The electrostatic force between a silicon probe and an alumina ceramic sample was measured, selecting tip-sample distance and applied voltage as variables that are easy to control. The fundamental characteristics of the electrostatic force were also investigated. The effects of applied voltage on the probe and the sample electrode were compared, and significant differences were found. We conclude that it is advisable to apply voltage on the probe. Then, from the phase difference obtained in AM-AFM, we derived the functional relationships between the absolute magnitude of electrostatic force and the distance under different voltages. The results show that the variation of electrostatic force with distance and applied voltage at the micro/nano scale are similar to those at the macroscopic scale. Electrostatic force decreases with increasing distance, but the decreasing rate gradually becomes slower and slower. On the other hand, the electrostatic force increases with increasing voltage and is basically proportional to the square of the applied voltage. These characteristics are applicable over a certain distance range. Beyond this range, the impacts of the distance and the voltage on the electrostatic force have a weakening trend.
